# Phase-change materials-based platforms for biomedicine

**DOI:** 10.3389/fbioe.2022.989953

**Published:** 2022-09-02

**Authors:** Biao-Qi Chen, Yu-Jing Pan, Da-Gui Zhang, Hong-Ying Xia, Ranjith Kumar Kankala

**Affiliations:** ^1^ Institute of Biomaterials and Tissue Engineering, Huaqiao University, Xiamen, China; ^2^ Fujian Provincial Key Laboratory of Biochemical Technology (Huaqiao University), Xiamen, China

**Keywords:** gatekeeper, temperature-controlled release, liposomes, core-shell architectures, phase-change materials

## Abstract

Recently, phase-change materials (PCMs) have gathered enormous attention in diverse fields of medicine, particularly in bioimaging, therapeutic delivery, and tissue engineering. Due to the excellent physicochemical characteristics and morphological characteristics of PCMs, several developments have been demonstrated in the construction of diverse PCMs-based architectures toward providing new burgeoning opportunities in developing innovative technologies and improving the therapeutic benefits of the existing formulations. However, the fabrication of PCM-based materials into colloidally stable particles remains challenging due to their natural hydrophobicity and high crystallinity. This review systematically emphasizes various PCMs-based platforms, such as traditional PCMs (liposomes) and their nanoarchitectured composites, including PCMs as core, shell, and gatekeeper, highlighting the pros and cons of these architectures for delivering bioactives, imaging anatomical features, and engineering tissues. Finally, we summarize the article with an exciting outlook, discussing the current challenges and future prospects for PCM-based platforms as biomaterials.

## Introduction

In recent times, tremendous advancements have resulted in the development of various advanced nanotechnology-based approaches for targeted delivery to diseased areas precisely with improved biodistribution and appropriate excretion profiles (Fan et al., 2017; Ramasamy et al., 2017). Although the impressive progress in pharmaceutics and materials science has resulted in the diverse nanocarriers with altered sizes and surface properties, the exploration of stimuli-responsive materials has garnered enormous attention, featuring reversible response to a specific stimulus, gating ability to avoid undesired release, highly conducive to load multiple drug payloads, and biodegradability, as well as biocompatibility (Mura et al., 2013). To satisfy these requirements and their subsequent translation, several efforts have been dedicated to using polymeric materials that respond to specific stimuli (receptors, biomarkers, and microenvironments) to formulate smart nanocarriers for precise therapy of the disease (Chen et al., 2018). Nonetheless, several attributes of multi-step preparation and low degradability-induced toxicity risks due to chemical modifications may hinder their applicability, limiting the subsequent translation to clinics.

**FIGURE 1:**
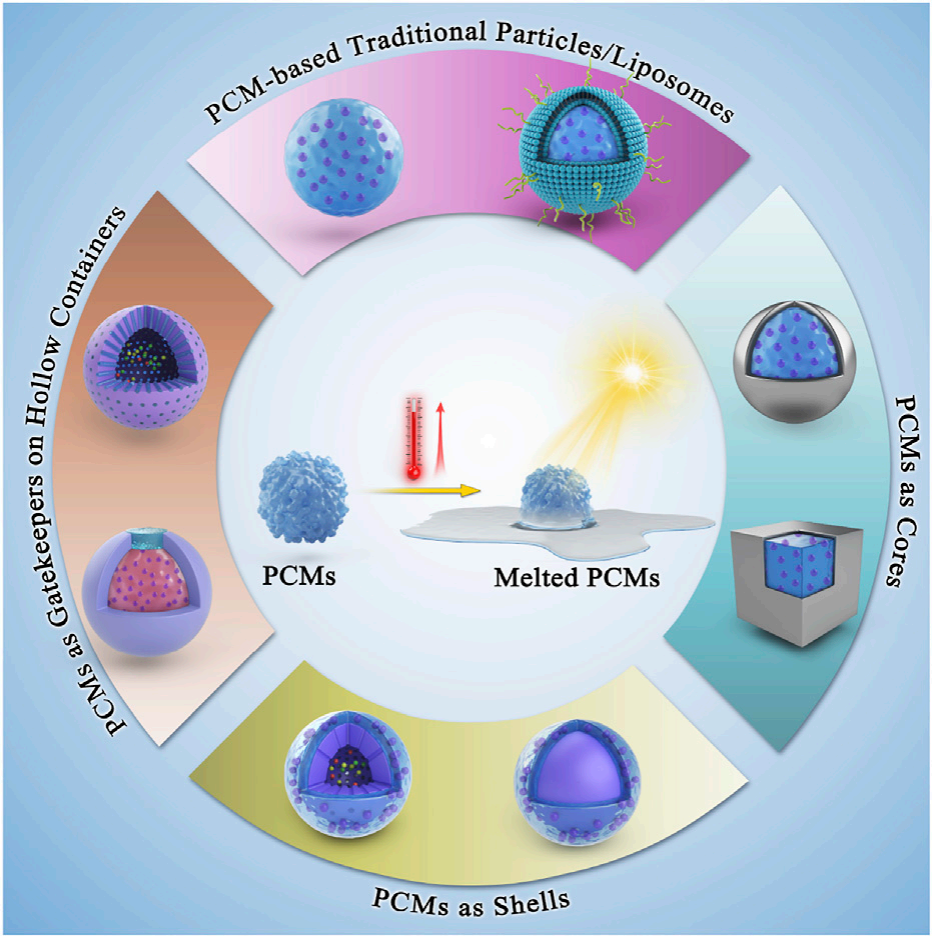
Schematic illustrating the conceptual design of various PCMs, in terms of positioning the PCMs and their successive nanoconjugates for biomedicine.

Phase-change materials (PCMs) with unique transition ability between solid and liquid states due to enormous latent fusion heat, have gained particular interest in thermal energy storage and solar energy applications (Dai et al., 2019; Ma et al., 2021). Among various phase transitions (i.e., solid-to-solid, solid-to-liquid, and liquid-to-gas), the solid-to-liquid changeover is often employed due to multiple features of the low transition temperature and high latent energy, as well as excellent thermal conductivity (Sun et al., 2019). These smart matrices encapsulate high drug payloads inside solid PCM and swiftly release them in response to a temperature upon transition from the solid-to-liquid phase (Fu et al., 2021). The classic examples include various thermo-responsive materials, such as natural fatty acids (lauric acid, LA, and stearic acid, SA) or fatty alcohols (1-tetradecanol), as well as their eutectic mixtures due to excellent biocompatibility/biodegradability, suitable melting point, chemical stability, and cost-effectiveness (Zhu et al., 2017a; Qiu et al., 2020). Due to their stable melting points of >37°C and satisfactory release rates, PCMs can be applied as biomaterials for promising therapeutic applications. Typically, photothermal conversion agents (PTCAs) and payloads are co-encapsulated in PCMs-based platforms to trigger light-assisted melting. Upon light irradiation, the platforms would be quickly heated up due to the photothermal effect of the encapsulated PTCAs (Liu Z. et al., 2020; Otaegui et al., 2020). Notably, if the local temperature is increased beyond the melting point, the platforms would melt, leading to the quick and on-demand release of the encapsulated payloads. Although several reviews have been published discussing the PCMs-based platform for biomedicine, the position of PCM in the drug delivery field has received tremendous attention recently. Therefore, a timely review of relevant research progress is of great significance for the continuous development of PCMs-based platforms. From a unique perspective of the PCMs-based platform architecture, in this mini-review, we systematically emphasize various platforms, such as traditional PCMs (liposomes) and their nanoarchitectured composites as thermo-responsive materials, including PCMs as core, shell, and gatekeeper, highlighting the pros and cons of these architectures for delivering bioactives, imaging anatomical features, and engineering tissues (Figure 1). Finally, we summarize the article with an exciting outlook, discussing the current challenges and future opportunities for PCM-based platforms as biomaterials.

## Phase-change materials-based platforms

### Phase-change materials-based traditional particles/liposomes

**FIGURE 2:**
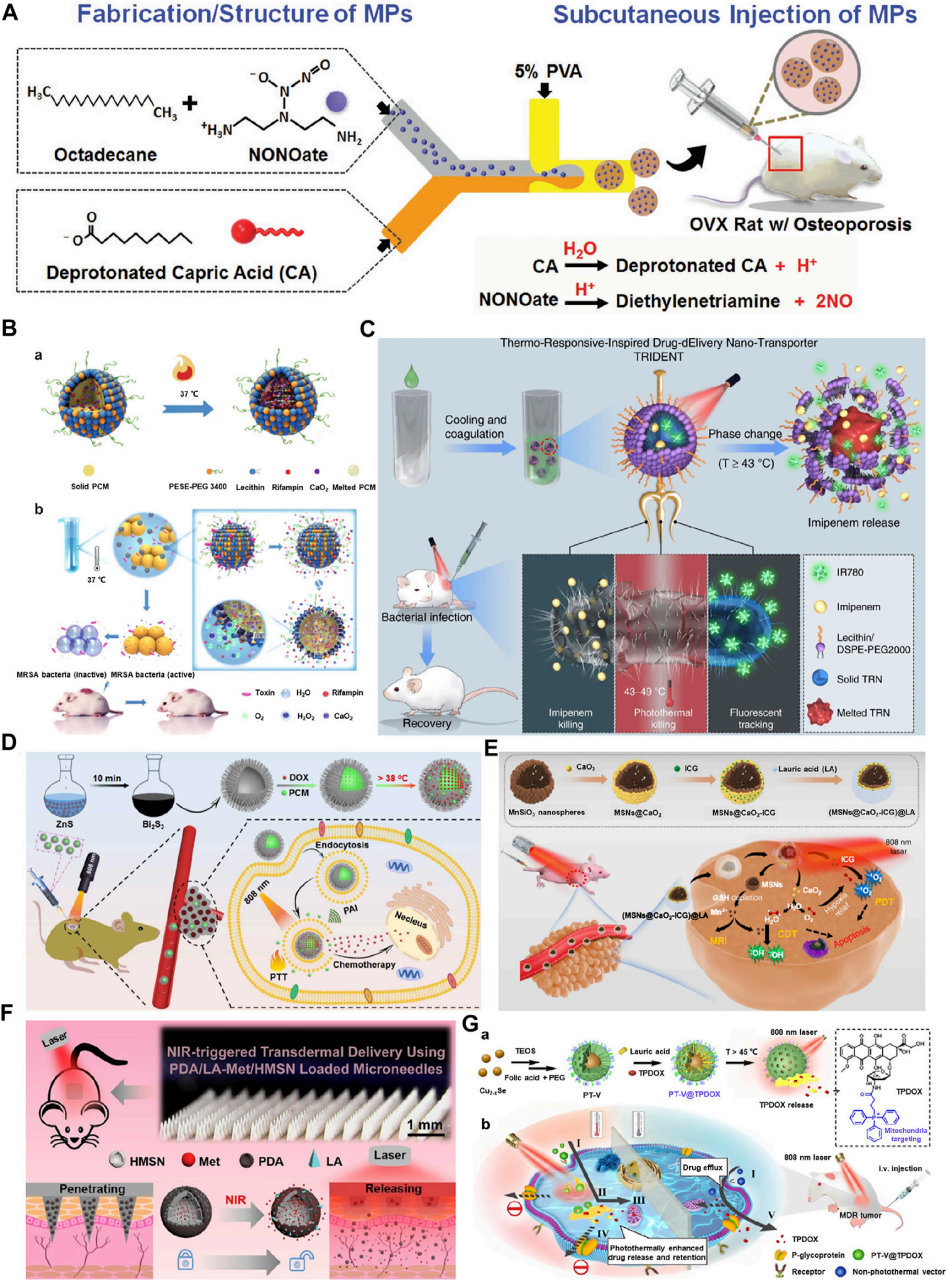
**(A)** Fabrication/structure of injectable microparticles (MPs). The MPs system is developed using a microfluidic device in an oil-in-water (O/W) single emulsion that consists of phase-change materials capric acid and octadecane and encapsulates NONOate. Reproduced with permission from Ref. (Lin et al., 2018). Copyright 2018, John Wiley and Sons. **(B)** Design and characterization of PCM-based liposome nanoreactors. (a) The solid PCM was dissolved in melted PCM at 37 °C. (b) The scheme of endogenous stimulus-powered antibiotic release from RFP-CaO_2_@PCM@Lec nanoreactors for bacterial infection combination therapy. Reproduced with permission from Ref. (Wu et al., 2019). Copyright 2019, Springer Nature. **(C)** Near infrared-activated nano-transporter (TRIDENT, also named IMP/IR780@TRN) for antibiotic-resistant bacteria killing. The prepared thermo-responsive-inspired drug-delivery nano-transporter is “melted” when the temperature rises above 43°C under the NIR irradiation, leading to the release of imipenem to the infected site. Reproduced with permission from Ref. (Qing et al., 2019). Copyright 2019, Springer Nature. **(D)** Schematic illustration of thermosensitive urchin-like Bi_2_S_3_ hollow microsphere as a carrier of DOX/PCM for photoacoustic imaging and photothermal-chemo therapy of tumors. Reproduced with permission from Ref. (Zhang C. et al., 2020). Copyright 2020, Elsevier. **(E)** The scheme of the fabrication process and therapeutic mechanism of thermo-responsive (MSNs@CaO_2_-ICG)@LA NPs for synergistic CDT/PDT with H_2_O_2_/O_2_ self-supply and GSH depletion. Reproduced with permission from Ref. (Liu C. et al., 2020). Copyright 2020, Springer Nature. **(F)** Schematics for preparation of metformin-loaded and PDA/LA-coated hollow mesoporous SiO_2_ nanocomposites and NIR-responsive release of loaded metformin on diabetic rats by the transdermal delivery method. Reproduced with permission from Ref. (Zhang et al., 2018). Copyright 2018, American Chemical Society. **(G)** Schematic illustration of (a) preparation of PT-V@TPDOX and (b) photothermally enhanced drug release and retention towards multidrug resistance cancer cells. I. Receptor-mediated endocytosis. II. Photothermally controlled drug release. III. Mitochondria targeting of TPDOX. IV. Inhibition of P-glycoprotein (P-gp) pathway. V. P-gp mediated drug efflux of TPDOX delivered by non-photothermal vector. Reproduced with permission from Ref. (Ji et al., 2021). Copyright 2020, Elsevier.

Among the classic examples of PCMs, natural fatty acids have gained enormous interest in the generation of traditional PCMs-based particles/liposomes due to their diversity, biocompatibility, biodegradability, abundance, and cost-effectiveness (Cao et al., 2021). For instance, capric acid (CA) and octadecane (OD) are lipophilic PCMs with melting points of 31 and 28°C, respectively, leading to phase change at body temperature and resulting in the leaching of their encapsulated guests. In an attempt to successfully deliver exogenous nitric oxide (NO) donors and address the short half-life of NO, injectable microfluidics-assisted microparticle (MP) systems were fabricated using the PCMs, CA, and OD. These PCM-based MPs as micellar depots successfully encapsulated NONOate, actively trapping and protecting the NO bubbles that are generated *in situ* (Figure 2A) (Lin et al., 2018). These PCMs could prevent the access of hemoglobin to NO bubbles and prolong half-life, resulting in sustained therapeutic function and retreating osteoporosis. In another case, a temperature-regulated system for the controlled release of nerve growth factor (NGF) to promote neurite outgrowth was reported (Xue et al., 2018). The system was based upon microparticles fabricated using a co-axial electrospray approach, with the outer solution containing PCMs (a mixture of LA and SA at a mass ratio of 4:1) and the inner solution encompassing NGF and a near-infrared (NIR) dye, indocyanine green (ICG). The controlled release system was evaluated for potential use in neural tissue engineering by sandwiching the microparticles between two layers of electrospun fibers to form a trilayer construct. Upon photothermal heating with a NIR laser, the NGF could be released on demand with well-preserved bioactivity to promote neurite outgrowth. This facile and versatile system could be readily applied to various biomedical applications by switching to different combinations of PCM, biological effector, and scaffolding material (Xue et al., 2020). Notably, the sensing temperature at the subcellular level is of great importance for understanding various biological processes. Recently, a novel organic fluorescent nanothermometer based on aggregation-induced emission (AIE) molecules and natural-derived PCMs was designed, and its application in non-invasive temperature sensing was explored (Xue et al., 2021a). First, a dual-responsive organic luminogen that could respond to the molecular state of aggregation and the environmental polarity was synthesized. Next, the natural saturated fatty acids with sharp melting points, and reversible, as well as rapid phase transitions were employed as the encapsulation matrix to correlate external heat information with the fluorescence properties of the luminogen. To apply the composite materials for biological application, colloidally dispersed nanoparticles were formulated by a technique based on *in situ* surface polymerization and nanoprecipitation. As anticipated, the resultant zwitterionic nanothermometer exhibited sensitive, reversible, reliable, and multiparametric responses to temperature variation within a narrow range around the physiological temperature (i.e., 37°C). Taking spectral position, fluorescence intensity, and fluorescence lifetime as the correlation parameters, the maximum relative thermal sensitivities were determined to be 2.15, 17.06, and 17.72%°C^−1^, respectively, which were much higher than most fluorescent nanothermometers.

Despite the successful encapsulation of the cargo, the particles made of fatty acids suffer from poor aqueous dispersibility, resulting in surface aggregation, which could be considerably addressed by adding amphiphilic molecules, such as phospholipids (Xue et al., 2021b). For instance, calcium peroxide (CaO_2_) and antibiotics were encapsulated in a eutectic mixture of fatty acids (4:1, SA, m. p. = 71.8–72.3 °C, and LA, m. p. = 45.7–46.2 °C) and coated with liposome (lecithin and DSPE-PEG3400) against bacterial infections. The release could happen sequentially in a series of steps, in which after bacteria contact the nanoreactors at 37 °C, anchored on the nanoreactor’s surface, form pores in the layer, entry of H_2_O molecules into the nanoreactors, resulting in the decomposition of formed H_2_O_2_ and driving antibiotic release (Figure 2B) (Wu et al., 2019). Similarly, versatile architectures referred to as TRIDENT (Thermo-Responsive-Inspired Drug-Delivery Nano-Transporter)-based on PCM using SA and LA were fabricated to address the synergistic effects of fluorescence monitoring and chemo-photothermal-based antimicrobial effectiveness against multidrug-resistant (MDR) bacteria. These hydrophobic TRIDENT PCMs encapsulated with a broad-spectrum antibiotic (imipenem, IMP) and IR780 and subsequently coated with lecithin and DSPE-PEG 2000 not only resulted in the NIR-assisted melting of the nanotransporter but also damaged the membrane facilitating the permeation, as well as interfering in the cell wall biosynthesis and enable bacterial death (Figure 2C) (Qing et al., 2019). In this regard, several PCMs-based on LA and SA, as well as oleic acid, were fabricated for nanotheranostics with the ability of hyperthermia-triggered spatiotemporally tunable drug release (Cai et al., 2021; Lai et al., 2022).

### Phase-change materials as cores

Despite the success, the PCMs sometimes may suffer from undesired degradation due to hypersensitivity, resulting in the unwanted leakage of encapsulated therapeutic cargo *in vivo*. To avoid the pre-degradation of fatty acid and subsequent pre-leakage of payload, PCMs and drugs were encapsulated in the micro-/nano-scale carriers as core substances (Zhang et al., 2022). These PCM cores facilitate the protection of therapeutic agents and execute their versatility in the appropriate circumstances. In a case, a eutectic fatty acid mixture of LA and SA with a melting temperature of 39°C and coloaded with doxorubicin (DOX) and ICG was encapsulated in silica-based nanocapsules using the site-selective deposition by templating with Au–PS Janus colloidal particles (Qiu et al., 2019). In another instance, Au nanocages (AuNCs) were encapsulated with the PCM (1-tetradecanol) and either hydrophobic or hydrophilic therapeutics, in which the PCM served as an inner gatekeeper to spatially control the NIR-triggered release in response to raising in temperature beyond the melting point (Moon et al., 2011).

Sonodynamic therapy (SDT), a non-invasive therapeutic strategy, offers enormous potential in treating solid tumors due to its high penetration depth (Bai et al., 2021). Nevertheless, the efficacy is limited due to hypoxia in solid tumors. In an attempt to address this issue, ultrasound-activated nanosystems based on the biodegradable hollow mesoporous organosilica nanoplatforms were developed by encapsulating ferrate (VI) and protoporphyrin IX, followed by PCM, LA deposition (Fu et al., 2019). The hydrogen peroxide and glutathione (GSH)-dependant oxygen production by ferrate (VI) species and subsequent ROS production by protoporphyrin-augmented SDT and intracellular Fenton chemistry, as well as ultrasound-assisted mild hyperthermia leading to phase change of LA, played a synergetic role in SDT-sensitized effects against solid hypoxic tumors. The low melting point of LA (44–46°C) endowed the temperature-sensitive control by the nanosystems over the diffusion of water and release of oxygen. In another instance, Bi_2_S_3_ hollow urchin-like nanostructures co-loaded with DOX and 1-tetradecanol with a melting point around 38 °C in the hollow cores for photoacoustic imaging and chemo-/photothermal therapy of tumors (Zhang C. et al., 2020). These composites facilitated the conversion of 808 nm NIR-assisted irradiation to heat energy, resulting in the triggered DOX release from the hollow containers after reaching the PCM melting point (Figure 2D). The tumor ablation efficiency, along with photoacoustic imaging and combined therapies, were systematically demonstrated *in vitro* and *in vivo*. Similarly, anticancer drugs and 1-tetradecanol were filled into the hollow magnetic nanoparticles for imaging-guided thermo-chemo combination cancer therapy. The system demonstrated a sensitive thermal response to the alternating current magnetic field for triggering switchable controlled drug delivery with a nearly “zero release” feature. More importantly, the system displays infrared thermal and magnetic resonance imaging properties for the image-guided cancer therapy (Li et al., 2015).

### Phase-change materials as shells

Considering the stability of the encapsulated therapeutics in a physiological environment, these PCMs can be employed to coat over highly sensitive molecules as shells. These PCM-based shells not only facilitate the protection of the encapsulated cargo but also enable their precise release through a thermo-responsive manner (Zhang S. C. et al., 2020). In a case, manganese silicate nanospheres (MSNs) supported by calcium peroxide (CaO_2_) and ICG were coated with the LA (MSNs@CaO_2_-ICG)@LA) for photodynamic (PDT)/chemodynamic (CDT) synergistic cancer therapy (Figure 2E) (Liu C. et al., 2020). The biocompatible and biodegradable LA with a melting point of 44–46°C on the surface was melted due to the NIR-assisted photothermal effect of ICG, in which the exposed CaO_2_ would react with water, generating H_2_O_2_ and O_2_, as well as accompanying the exposure of MSNs towards Fenton-like agent Mn^2+^ for H_2_O_2_-supplementing CDT and MRI-guided synergistic therapy. In an attempt to explore gas therapy with negligible side effects, Zhu and colleagues developed a new type of multi-shell nanoparticles (CuS@SiO_2_-l-Arg@PCM-Ce6, CSLPC), in which the PCM wax-sealed profile of the encapsulated Ce6 would be released with the NIR-II-assisted, CuS-triggered photothermal effect in the tumor site (Zhu et al., 2021). In addition, the released l-Arg was oxidized to generate NO for gas therapy, resulting in the synergistic targeted tumor therapy. Similarly, multifunctional nanosystems based on hollow mesoporous organosilica nanoparticles (HMONs) deposited with CuS were generated for the dual stimuli-responsive drug delivery (Chen et al., 2020). These composites coated with 1-tetradecanol substantially facilitated the avoidance of drug leakage and improved CuS-based NIR-assisted temperature-controlled release of encapsulated DOX cargo for chemo- and photothermal therapy. In another similar instance, hollow mesoporous SiO_2_ nanoparticles (HMSNs) were coated with PCM (polydopamine (PDA) as photothermal conversion agent/LA, mp ≈ 44–46°C) for the successful delivery of metformin through the NIR-responsive poly (vinylpyrrolidone) microneedle (MN) system (Figure 2F) (Zhang et al., 2018). These MNs for transdermal delivery facilitated the triggering effects of PCM by NIR-responsiveness after being inserted in the skin, leading to the release of the encapsulated cargo from MNs.

### Phase-change materials as gatekeepers on hollow containers

Similar to enclosing various therapeutics in the PCM as shells to protect them from pre-leakage, the PCMs can be specifically utilized as gatekeepers on the pores of various inorganic porous architectures. These PCM-based gatekeepers facilitate the protection of enclosed therapeutic cargo and enable their precise release in a specific environment (Hussain and Guo, 2019; Li et al., 2021). It should be noted that the precise selection of PCM depends on the application and the environment that could precisely transform the PCM. Although DOX is the most preferred anticancer molecule in clinics, it is often suffered from MDR efflux, hindering its performance efficacy. In an attempt to address these aspects, TPDOX is encapsulated in the pores of mesoporous silica, please see (Figure 2G). The porous silica was filled with LA (melting point is 45 °C) along with TPDOX in the form of small-sized particles with an average diameter of 40 nm. The NIR (808 nm) laser-assisted melting of LA facilitated the release of TPDOX, significantly inhibiting drug efflux and enabling antitumor therapy (Ji et al., 2021). In another case, You and group fabricated a 1-tetradecanol-based ICG-loaded CuS@mSiO_2_ nanoplatform (CuS@mSiO_2_-TD/ICG) (You et al., 2017). The NIR (808 nm)-absorbing ICG in mesopores facilitated the melting of PCM (1-tetradecanol) gatekeepers, resulting in the CuS@mSiO_2_-assisted PTT and simultaneously ICG-based PDT/PTT effects. The PCM, 1-tetradecanol, offers a reversible change in its physical states at a narrow temperature range, in which it exists as solid in the body temperature but melts at just above it (Tm = 39°C). These observations showcase that the porous cavities are opened rapidly above the body temperature after exposure to the heating source.

In most instances, the PCMs are often based on fatty acids or fatty alcohols, in which the encapsulated drug can be released by substantially melting the PCM by raising the temperature beyond its melting point (Zhu et al., 2017b). However, the release is substantially dependent on the encapsulated PCM species, which could be limited to specific cargo. The precise control over the release kinetics can be altered by regulating the melting point of PCMs, which can be achieved by the composition of different PCM species with a mixture of 1-tetradecanol (at 38°C) and LA (at 44°C) at different ratios (Hyun et al., 2013).

## Conclusions and perspectives

In summary, this article has reviewed the recent advances in the development of PCM-based platforms for biomedical applications. Due to their specific physicochemical attributes, these PCMs and their composites (cores, shells, and gatekeepers) have shown excellent prospects in diverse biomedical applications. Despite the success in exploring the characteristics, some unwanted characteristics of PCMs during the phase transition must be altered, for instance, undercooling, volume expansion, low thermal conductivity, and phase separation. In addition, various necessities must be comprehensively considered to meet the application requirements for expanding the scope of PCMs for biomedical applications. Several application principles are required to be addressed according to the application requirements, such as appropriate phase transition temperature and latent heat, suitable chemical stability during the phase change, biosafety, and ease of synthesis using cost-effective precursors, as well as eco-friendly techniques.

Despite the enormous progress, several key features are required to be strictly optimized for their clinical translation. 1) The foremost requirement is the morphological attributes concerning the particle size and pore diameters in the case of mesoporous architectures, as well as shell thickness in the core-shell structures. It should be noted that these morphological features influence the thermal characteristics of PCMs. 2) Efforts to alter the PCM surfaces and regulate the mesoporous characteristics are required further to improve the translation of the PCMs. 3) Similarly, the temperature changes and their effect, along with the mechanistic views, are yet to be resolved. Although several studies have explored the temperature-related PCM conversion and their subsequent synergistic effects on cancer therapy, it is required to investigate the related viewpoints in various other ailments. 4) The biosafety of these PCMs and their composites must be necessary to explore comprehensively, right from the *in vitro* to *in vivo* assessments.

Among the aforementioned challenging tasks, the predominant efficacy-related issue is that realizing the phase transition of PCM materials in deep human tissues remains further studied due to the limited tissue penetration depth of light. To a considerable extent, using ultrasound, X-rays, or magnetic fields to stimulate heat production may help solve these problems. In recent years, catalyzing or *in situ* generations of active substances at the lesion site for treating diseases is an important research direction for precision therapy. Applying PCMs to coat catalysts or substrates, release them quickly after reaching the lesion site, and initiate relevant chemical reactions to treat diseases may be an important research direction for PCMs in the future. In summary, the current review explored the detailed insights of the relevant communities working on PCMs and their composites, which could be applied to biomedical applications.
